# Exploring the Associations Between Childhood ADHD Symptoms, Higher Education and Depressive Symptoms

**DOI:** 10.1192/bjo.2026.11081

**Published:** 2026-06-30

**Authors:** Natalie Kravtsov

**Affiliations:** Essex Partnership University NHS Foundation Trust (EPUT), Chelmsford, United Kingdom

## Abstract

**Aims:**

Young people with Attention-Deficit Hyperactivity Disorder (ADHD) may struggle with mental health during university, yet few studies have looked at how university affects their mental health in adulthood. This study investigated the association between childhood ADHD symptoms and depressive symptoms at age 25, as well as the association with university graduation, and examined to what extent university graduation explains the association between ADHD symptoms and depressive symptoms.

**Methods:**

ALSPAC (Avon Longitudinal Study of Parents and Children) is a cohort study that collected information on children born in the 1990s. 1,574 individuals were included in analyses who had data available on ADHD hyperactivity symptoms at age nine, depressive symptoms at age 25, university graduation at age 21, 22, or 23, and confounders. Regression modelling and mediation analysis were used to understand the relationships between these factors.

**Results:**

Childhood ADHD symptoms, being in the top category of 7+ points on the Strengths and Difficulties Questionnaire (SDQ) at age nine, was associated with increased depressive symptoms (b=1.72, 95% CI: 0.34–3.10) and decreased likelihood of completing university (OR=0.42, 95% CI=0.25–0.69, p*=*0.001). University graduation mediated 11.7% of the relationship between childhood ADHD symptoms and depressive symptoms.

**Conclusion:**

Childhood ADHD symptoms are negatively associated with university completion. There was only weak evidence for an association between childhood ADHD symptoms and adult depressive symptoms being mediated by university graduation. This highlights the importance of continuing investment in university mental health support and earlier support for children.

